# Case Report: Pediatric palisaded neutrophilic and granulomatous dermatitis without systemic diseases treated with sulfasalazine

**DOI:** 10.3389/fmed.2025.1670091

**Published:** 2025-10-31

**Authors:** Qiuyang Guo, Yumeng Wang, Qiuyi Han, Jinxiang Yang, Jiayan Zhang, Guofang Li, Yijun Yang, Ruhong Cheng, Zhirong Yao

**Affiliations:** ^1^Dermatology Center, Xinhua Hospital, Shanghai Jiao Tong University School of Medicine, Shanghai, China; ^2^Department of Dermatology, Xinhua Hospital, Shanghai Jiao Tong University School of Medicine, Shanghai, China; ^3^Institute of Dermatology, Shanghai Jiao Tong University School of Medicine, Shanghai, China

**Keywords:** palisaded neutrophilic and granulomatous dermatitis, neutrophilic dermatosis, sulfasalazine, pediatric, cutaneous-limited

## Abstract

Palisaded neutrophilic and granulomatous dermatitis (PNGD) is a rare neutrophilic dermatosis strongly associated with systemic conditions. Pediatric cases with exclusive cutaneous involvement are exceptionally rare. Current treatment options for PNGD include oral dapsone, hydroxychloroquine, and intralesional or systemic corticosteroids. Here we report a 6-years-old girl with skin-limited PNGD, presenting symmetric annular plaques on limb extensor surfaces and atypical lesions on the face, palms, and buttocks. Significant lesion resolution was achieved after 2 months of oral sulfasalazine therapy (25 mg/kg/day in divided doses), with only mild abdominal discomfort. To our knowledge, this represents the first successful use of sulfasalazine for PNGD, providing a dapsone-sparing alternative for pediatric PNGD.

## Introduction

Palisaded neutrophilic and granulomatous dermatitis (PNGD) is a rare neutrophilic dermatosis that can occur in individuals of all ages, although reports of PNGD in children are limited (1–4). The male-to-female ratio is approximately 1:3, which may be attributed to the fact that PNGD is most commonly associated with systemic conditions such as connective tissue diseases, inflammatory arthritis, lymphoproliferative disorders, or infections (5–8), with few reports of PNGD occurring without underlying disorders (7, 9–11). Asymptomatic erythematous papules symmetrically distributed on the extensor surfaces of the upper limbs are the typical clinical presentation of PNGD, most commonly affecting the elbows and fingers (12). PNGD may also present as patches, nodules, linear cords, and plaques (6, 12). Different stages of the disease exhibit varying histopathological features, with early manifestations typically characterized by leukocytoclastic vasculitis, neutrophilic infiltration, nuclear debris, and amorphous basophilic substances present in the dermis; whereas later stages are mainly marked by palisaded granulomas and degenerative collagen (5). PNGD can be managed by controlling underlying systemic diseases, and specific treatment options for PNGD include oral dapsone, hydroxychloroquine, and intralesional or systemic use of corticosteroids (13). Spontaneous remission may also occur in some cases of PNGD. Here, we report a child with PNGD without associated systemic disease who achieved resolution of the rash following oral sulfasalazine treatment. To our knowledge, this is a rare report of skin-limited pediatric PNGD and the first report of sulfasalazine treatment for PNGD.

## Case report

A 6-years-old girl presented to our hospital with multiple papules on her face, limbs, and buttocks for 6 months. The rash initially appeared on the extensor surfaces of both elbows and knees, without significant pruritus or pain. After treatment with topical corticosteroids and tacrolimus, the lesions failed to improve. According to the guardian, the patient had no history of systemic diseases or medication use, and the skin lesions had shown a tendency for spontaneous cyclical resolution prior to our evaluation. Throughout the course of illness, she remained afebrile and did not develop cough, chest pain, recurrent oral ulcers, significant arthralgia, or other systemic symptoms.

**FIGURE 1:**
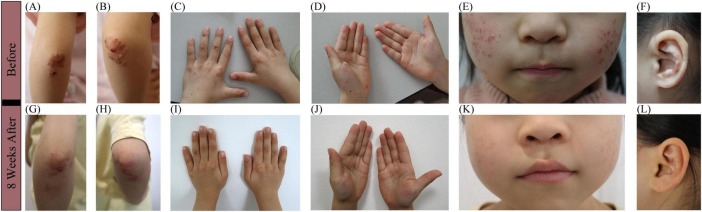
Clinical manifestations before and after 2-months sulfasalazine treatment. **(A,B)** Firm erythematous-to-yellow papules and annular, well-circumscribed plaques with raised borders on the extensor surfaces of both elbows, with central umbilication and surface scaling. **(C,D)** Firm, umbilicated papules with central crusting were observed on the dorsal hands, overlying the metacarpophalangeal and interphalangeal joints, and involving both palms. **(E,F)** Multiple papules and pustules on the bilateral cheeks and auricles. **(G–L)** Resolution of lesions after 2-months sulfasalazine treatment, with residual post-inflammatory hyperpigmentation.

Physical examination showed firm erythematous-to-yellow papules and annular, well-circumscribed plaques with raised borders on the extensor surfaces of both elbows (Figures 1A, B) and knees, as well as on the buttocks and posterior thighs, with central umbilication and surface scaling. Scattered firm umbilicated papules with central crusting were observed on the dorsal aspects of the hands at the metacarpophalangeal and interphalangeal joints (Figure 1C), as well as on both palms (Figure 1D) and ankles. Multiple papules and pustules were also noted on the bilateral cheeks and auricles (Figures 1E, F).

**FIGURE 2:**
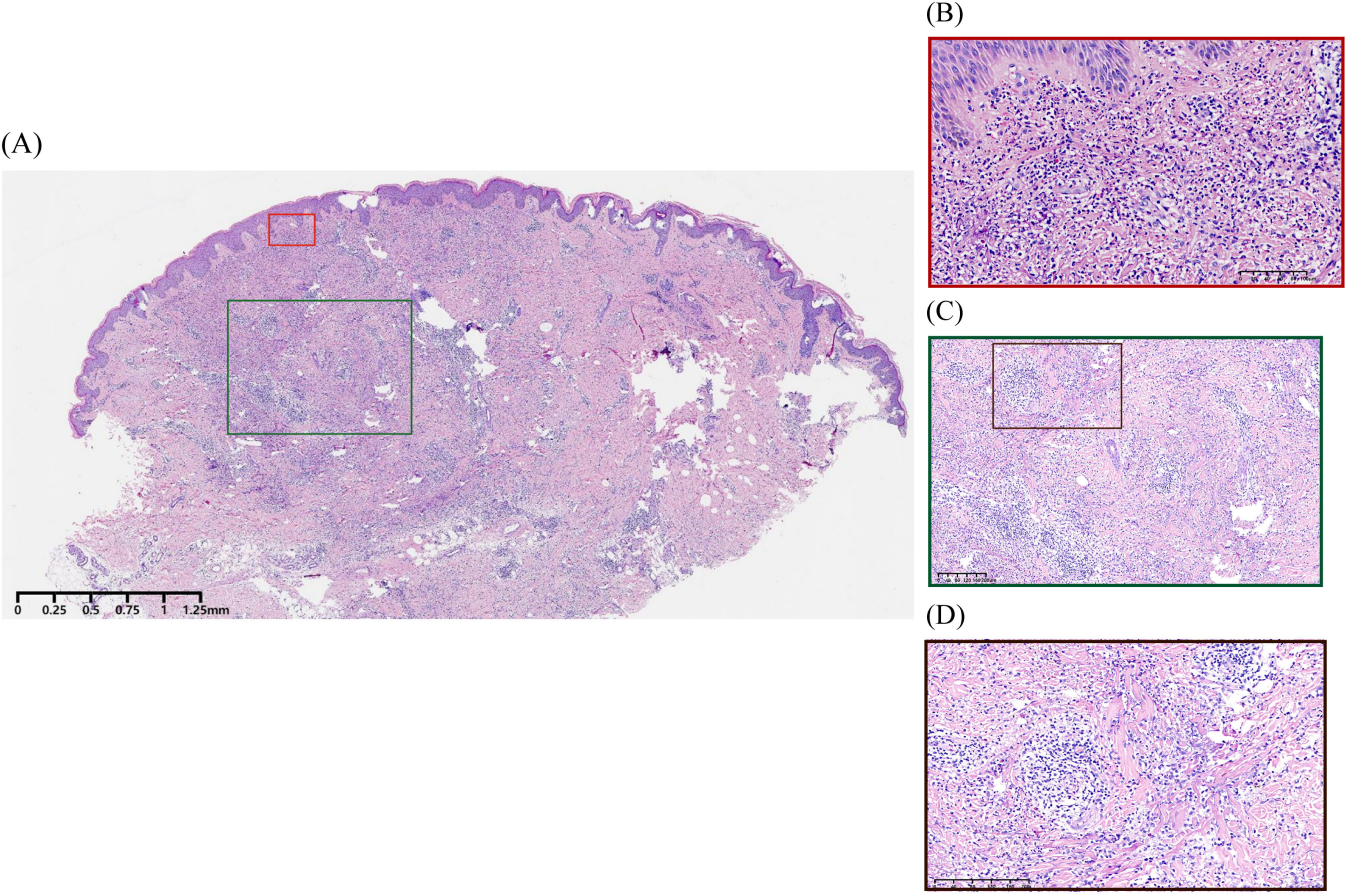
Histopathological examination of the patient. **(A)** Hyperkeratosis, mild irregular epidermal proliferation, and infiltration of neutrophils and lymphocytes in the dermis (H&E, ×67.5). **(B)** Leukocytoclastic vasculitis characterized by destruction of vascular wall structure, swelling of endothelial cells with neutrophilic infiltration, and extravasation of red blood cells accompanied by nuclear dust, without notable fibrin deposition in the vascular walls (H&E, ×900). **(C,D)** Palisading granulomatous inflammation surrounding degenerated collagen (H&E, ×337, ×674).

Urinalysis and stool routine tests showed no abnormalities. All laboratory test results were within normal ranges, including C-reactive protein (CRP), complete blood count with differential, erythrocyte sedimentation rate (ESR), liver and kidney function tests, complement testing, anti-streptolysin O (ASO) titer, T-SPOT assay for tuberculosis infection, hepatitis serology markers, as well as autoimmune antibody screening (ANA, anti-dsDNA, anti-Smith, anti-SSA, anti-SSB, anti-Scl-70, anti-Jo-1, anti-histone, and anti-nucleosome) and rheumatoid factor. Abdominal ultrasound and plain chest radiography revealed no abnormalities. Histopathological examination of an annular plaque on the buttocks revealed infiltration of neutrophils and lymphocytes in the dermis, leukocytoclastic vasculitis (Figures 2A, B), and dermal granulomata featuring central zones of degenerated collagen surrounded by histiocytes arranged in a palisading pattern (Figures 2C, D).

Based on the above information, this girl was diagnosed with PNGD without associated systemic disease. Due to the difficulty in obtaining dapsone in China, oral sulfasalazine was started at 25 mg/kg/day in two divided doses, with no concurrent topical therapy administered. The caregivers reported noticeable flattening of widespread dermatological lesions within 2 weeks of treatment commencement, with newly emerging lesions demonstrating rapid resolution within 48 h. Now, after a 2-months oral maintenance treatment, the majority of her original lesions flattened (Figures 1G–L), leaving only post-inflammatory hyperpigmentation. The patient has experienced only occasional mild abdominal pain, with no other significant side effects observed. Routine blood tests and autoimmune antibodies are being regularly monitored.

## Discussion

To the best of our knowledge, this is the first case of PNGD treated with sulfasalazine, providing valuable clinical treatment experience for children with rare PNGD who show no evidence of systemic disease.

The diagnosis of PNGD is based on characteristic clinical manifestations and histopathological features. PNGD typically manifests as symmetrical red papules on the extensor surfaces of the elbows and fingers, but can also extend to non-classic locations such as the legs, cheeks, nose, and scalp (6, 7). In our case, the patient presented with the characteristic symmetric distribution of papules on the elbows, dorsal hands, knees, and ankles, accompanied by umbilicated lesions or crusting. She also exhibited PNGD lesions in less common locations, including the cheeks, auricles, buttocks, posterior thighs, and palms. Notably, there is currently no evidence of malignancy, infection, or a definable connective tissue disease. The skin biopsy showed neutrophilic infiltration and leukocytoclastic vasculitis, with palisaded granulomatous changes and collagen degeneration. These findings support the diagnosis of cutaneous-limited PNGD.

The differential diagnosis includes granuloma annulare, erythema elevatum diutinum, rheumatoid neutrophilic dermatitis (RND), interstitial granulomatous dermatitis (IGD), and interstitial granulomatous drug reaction (IGDR) (5, 8, 13). Granuloma annulare can also present as a palisading granuloma, but it cannot account for the prominent neutrophilic dermal infiltration observed in our case, while erythema elevatum diutinum does not explain the palisading dermal cell infiltration as well as PNGD. RND is a rare neutrophilic skin condition that has been associated with rheumatoid arthritis (RA) and does not exhibit vasculitis or palisading granulomatous pathology (14). PNGD, IGD, and IGDR–collectively referred to as reactive granulomatous dermatitis (RGD)–share overlapping clinical and histological features. However, IGD commonly presents as linear subcutaneous cords on the proximal trunk, typically lacking vasculitis histologically (5, 8). The “floating sign” (histiocytes surrounding fragmented collagen) is a characteristic pathological feature of IGD. In contrast, IGDR can be distinguished from PNGD by a history of drug exposure and histological findings of vacuolar interface dermatitis, dermal eosinophilic infiltration, and lymphocytic atypia.

The majority of reported PNGD treatments primarily target concomitant systemic diseases (15, 16). In the case presented, the patient exhibited no clinical evidence of underlying systemic disease, and histopathological examination revealed neutrophilic infiltration. Based on these findings, we elected to avoid unnecessarily aggressive therapies including systemic corticosteroids (with associated risks of immunosuppression, metabolic disturbances, and pediatric growth suppression) and hydroxychloroquine (with potential retinopathy risk), opting instead for neutrophil-targeted treatment. Current evidence indicates that oral dapsone, a sulfonamide antibiotic that inhibits myeloperoxidase activity in neutrophils, may demonstrate therapeutic efficacy for PNGD (5, 17). Due to the limited availability of dapsone in China, we opted for oral sulfasalazine as an alternative treatment. Sulfasalazine, which consists of sulfanilamide and 5-aminosalicylic acid, is metabolized in the gastrointestinal tract to yield sulfanilamide, a sulfonamide antibiotic that shares a similar structure and possesses comparable antibiotic and anti-metabolic properties to dapsone, including the ability to inhibit myeloperoxidase. Sulfasalazine has also been proven to inhibit the synthesis of chemotactic lipids by neutrophils (18), thus attenuating neutrophil chemotaxis and, thereby, reducing subsequent tissue damage. Clinical evidence has demonstrated sulfasalazine’s therapeutic efficacy comparable to dapsone for certain neutrophilic dermatoses, with a favorable safety profile (17, 19). We discontinued topical medications when initiating sulfasalazine therapy given the documented limited efficacy of topical treatments for PNGD (5), absence of significant infection or exudation in the lesions, and the patient’s demonstrated failure to respond to months of prior topical corticosteroids and tacrolimus treatment.

By contextualizing our findings within the scarce pediatric PNGD literature, this report reinforces the heterogeneous nature of the disease (Table 1). It expands the known demographic to include younger, systemically healthy children, documents a broader spectrum of cutaneous manifestations, and proposes oral sulfasalazine as a novel, effective, and well-tolerated systemic therapy.

**TABLE 1:** Comparative clinical features, histopathology, treatment and outcomes of pediatric PNGD cases.

Category	Our case	Germanas et al. (3)	Biswas et al. (4)	Hunt et al. (2)	Nguyen et al. (1)
Sex/age	F/6	F/12	F/10	F/14	F/15
Associated systemic diseases	None	SLE	Type I diabetes, celiac disease	pANCA-positive glomerulonephritis	Juvenile idiopathic arthritis, psoriasis
Clinical features	Papules, plaques with central umbilication on face, elbows, knees, buttocks, hands (including palms), and ankles.	Annular plaques on neck, arms, flank, abdomen, and thighs.	Erythematous papules in an annular and linear pattern on feet and ankles.	Umbilicated papulonodules on elbows, knees, buttocks, hands, and ears.	Indurated papules and nodules on posterior arms and thighs.
Histopathology	Leukocytoclastic vasculitis and palisaded granulomas.	Neutrophils, histiocytes, lymphocytes; mucin deposition.	Palisaded granulomas with neutrophils and vasculitic reaction pattern.	Ranged from dense neutrophilic infiltrate to well-developed palisaded granulomas over time.	Diffuse neutrophilic infiltrate with ill-defined granulomas.
Treatment	Oral sulfasalazine	IV methylprednisolone and cyclophosphamide (for SLE)	None (resolved with glycemic control)	Dapsone, systemic prednisone	Topical triamcinolone
Outcome	Near-complete clearance after 2 months.	Recurrent, lesions resolved with each cycle of immunosuppressive therapy.	Spontaneous resolution; recurrence improved with glycemic control.	Recurrence after discontinuation of dapsone.	Recurrent, lesions appeared after each etanercept injection but improved with topical therapy.

F, female; SLE, systemic lupus erythematosus; pANCA, perinuclear antineutrophil cytoplasmic antibody; IV, intravenous.

Although oral sulfasalazine has been investigated and shown clinical efficacy in the RGD disease spectrum (20), RGD can be classified into four distinct histopathological subtypes: neutrophil-predominant, histiocyte-predominant, eosinophil-predominant, and mucinous type (5). As PNGD represents the neutrophil-predominant variant and no prior studies have documented sulfasalazine use specifically for this histopathological subtype, our case appears to be the first reported instance of PNGD successfully treated with sulfasalazine, thereby providing a valuable therapeutic reference for managing pediatric PNGD without systemic involvement. Nevertheless, it is important to acknowledge the limitation that these findings are derived from a single case report, and further studies are warranted to validate the efficacy of sulfasalazine in this specific context.

It is noteworthy that although there is currently no evidence of systemic involvement, PNGD can be a precursor to systemic disease that could manifest years later (21). Given the current lack of reports on pediatric PNGD without systemic conditions, we should emphasize the importance of regular monitoring of relevant indicators and maintaining proper follow-up.

## Data Availability

The original contributions presented in this study are included in this article/supplementary material, further inquiries can be directed to the corresponding authors.
